# Effectiveness and cost-effectiveness of a self-management group program to improve social participation in patients with neuromuscular disease and chronic fatigue: protocol of the Energetic study

**DOI:** 10.1186/s12883-015-0314-4

**Published:** 2015-04-19

**Authors:** Yvonne Veenhuizen, Edith HC Cup, Jan T Groothuis, Jan CM Hendriks, Eddy MM Adang, Baziel GM van Engelen, Alexander CH Geurts

**Affiliations:** Department of Rehabilitation, Donders Centre for Neuroscience, Radboud University Medical Centre, Nijmegen, Netherlands; Department for Health Evidence, Radboud Institute for Health Sciences, Radboud University Medical Centre, Nijmegen, Netherlands; Department of Neurology, Donders Centre for Neuroscience, Radboud University Medical Centre, Nijmegen, Netherlands

**Keywords:** Neuromuscular disease, Facioscapulohumeral muscular dystrophy (FSHD), Inclusion body myositis (IBM), Mitochondrial myopathy, Self-management, Participation, Effectiveness, Randomized controlled trial, Study protocol, Energy conservation

## Abstract

**Background:**

Chronic fatigue is present in more than 60% of the patients with a neuromuscular disease and can be their most disabling symptom. In combination with other impairments, fatigue often results in low levels of physical activity and decreased social participation, leading to high societal costs. ‘Energetic’ is a self-management group program aimed at improving social participation, physical endurance and alleviating fatigue in these patients. The primary aim of this study is to evaluate the effectiveness and cost-effectiveness of the Energetic program.

**Methods/Design:**

A multicentered, assessor-blinded, two-armed randomized controlled trial is conducted with evaluations at inclusion and four, seven and fifteen months later. The study includes patients with a neuromuscular disease and chronic fatigue and, when present, their caregivers. The participants are randomized (ratio 1:1) to either an intervention group, receiving the Energetic program, or a control group, receiving usual care (i.e., no specific intervention). The Energetic program covers four months and includes four modules: 1) individually tailored aerobic exercise training; 2) education about aerobic exercise; 3) self-management training in applying energy conservation strategies; and 4) implementation and relapse prevention in daily life. Two months after cessation of the program a booster session is provided. The primary outcome is the perceived performance score of the Canadian Occupational Performance Measure (COPM). Secondary outcomes include the COPM-satisfaction score, and measures of fatigue, physical endurance, activity engagement, mood, and self-efficacy. Caregiver burden is also evaluated as a secondary outcome. Health-related quality of life and medical and societal costs are assessed to estimate cost-effectiveness of the program.

**Discussion:**

The Energetic study is the first randomized controlled trial to evaluate the effectiveness and cost-effectiveness of a combined physical and self-management group training program for improving social participation, physical endurance and alleviating fatigue in patients with neuromuscular diseases. It will generate new insights in (cost-)effective rehabilitation strategies for these incurable conditions.

**Trial registration:**

Clinicaltrials.gov NCT02208687.

## Background

Chronic fatigue is present in more than 60% of patients with a neuromuscular disease (NMD), including facioscapulohumeral muscular dystrophy (FSHD), inclusion body myositis (IBM) and mitochondrial myopathies (MM), and can be their most prominent and disabling symptom [1,2]. In combination with muscle weakness, enhanced fall risk, and possible cardiopulmonary involvement, fatigue often results in low levels of physical activity and decreased social participation, which in turn leads to high societal costs. Compared to other neurological conditions, NMDs have the highest estimated costs of €30.000,- per person per year, with an estimated prevalence between 1:20.000 to 1:424 persons [3,4]. These include direct health costs related to treatment and rehabilitation, indirect costs related to low employment rates due to work absence or early retirement, and non-medical costs for social services, assistive devices and informal care [5].

In a recent randomized controlled trial, we have provided evidence that aerobic exercise training and cognitive behaviour therapy can both alleviate severe chronic fatigue in patients with FSHD [6]. Both interventions were based on a theoretical model of chronic fatigue in NMD, in which muscle strength, level of self-reported physical activity, sleep disturbances and pain were all associated with fatigue and, ultimately, with social participation [7]. Based on the first research results and experiences in patients with FSHD, we developed a multidisciplinary rehabilitation program to accommodate the clinical urge felt by many (other) patients with NMD and chronic fatigue. This rehabilitation program was called ‘Energetic’ and combines aerobic exercises and energy-conservation strategies in a self-management group program to improve social participation and physical endurance and alleviate fatigue. The program covers four months and includes four modules: (1) aerobic exercise training, (2) education about aerobic exercise, (3) self-management training in applying energy conservation strategies, and (4) implementation and relapse prevention in daily life. In the development of the program, we have acknowledged the research priorities identified by patients with NMD and healthcare professionals [8], and implemented the available clinical evidence regarding exercise training in NMD [6,9,10], fatigue management [11,12] and self-management training [13]. The following dimensions of self-management are addressed in the Energetic program; (1) medical management (e.g., regarding medication, diet or exercise (2) role management (regarding meaningful social roles), and (3) emotional management (dealing with emotions such as fear or depression) [13]. Self-management programs provide patients with the necessary knowledge, skills, and confidence (‘self-efficacy’) to manage life with a chronic illness and prepares patients to collaborate with their healthcare professionals and the healthcare system [13].

Although the Energetic program was well appreciated by participants already from the beginning, it has not yet been formally evaluated. So far, we only conducted an uncontrolled pilot study with pre-post measurements in 13 patients with a neurological condition (77% NMD) showing significant improvement in social participation based on the Canadian Occupational Performance Measure (COPM) and a significant decrease in fatigue as assessed with the Checklist Individual Strength - subscale Fatigue (CIS-Fatigue) [14]. These promising results formed the basis to conduct the present larger randomized controlled trial of the (cost-) effectiveness of the Energetic program in various forms of NMD, specifically FSHD, IBM and MM. In this study, the hypothesis is tested that the Energetic program results in improved social participation, better physical endurance and less fatigue in patients with NMD and severe chronic fatigue compared to no specific intervention (‘usual care’). Furthermore, we expect that these improvements will ultimately lead to a reduction in medical and societal costs.

## Methods/Design

**Figure 1:**
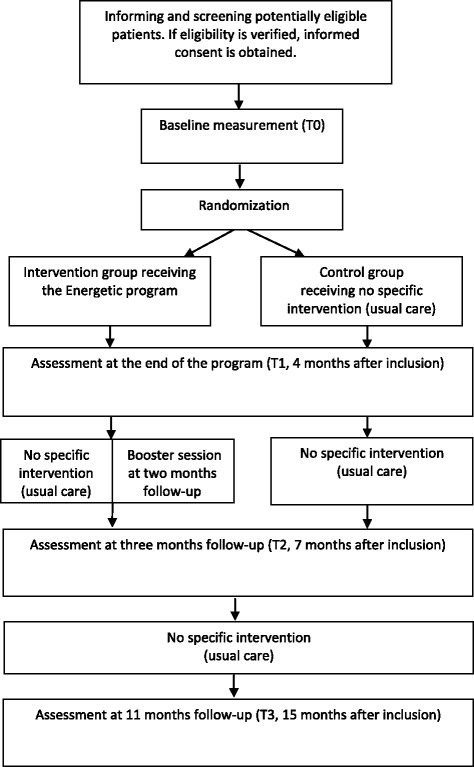
Flow chart of the Energetic study.

A multicentered, assessor-blinded, two-armed randomized controlled trial is conducted, in which eligible patients are randomized in a 1:1 ratio to either an intervention group, receiving the Energetic program, or a control group, receiving no specific intervention (‘usual care’). Randomization is based on a computerized minimization algorithm with the following minimization factors: gender, work (work ≥ no work), diagnosis (FSHD, IBM, MM, ≥ other NMD). Data will be collected at inclusion (before randomization, T0), at the end of the program (4 months after inclusion, T1), at three months follow-up (7 months after inclusion, T2), and at eleven months follow-up (15 months after inclusion, T3) (see Figure 1). Fifteen months after inclusion, the control group is offered participation in the Energetic program, but this post-study intervention period is not monitored.

All outcomes are assessed by blinded and independent occupational therapy research assistants. At the beginning of each assessment, participants are instructed not to reveal their group allocation to the assessor. After each assessment, blinding of the assessors is evaluated by asking them to indicate their idea of group assignment for each patient. Data entry is executed by the (blinded) research assistant. Adverse events or irregularities affecting protocol adherence are registered by the primary researcher (YV). Full ethical approval has been granted by the medical ethical committee of Arnhem-Nijmegen (NL47624.091.14) and all participating centers granted (ethical) approval to participate.

The Energetic study has been registered at clinicaltrial.gov (NCT02208687).

### Setting

Patients are recruited at either the departments of Rehabilitation, Neurology or General Internal Medicine of the Radboud University Medical Centre or at a major regional rehabilitation center (RMC Groot Klimmendaal). In addition, the Dutch NMD patient association ‘Spierziekten Nederland’ facilitates patient recruitment by posting information about the study on their website, in their magazine, and by sending mails to the specific patient groups (FSHD, IBM, MM). Based on patient preference, the Energetic program is offered and evaluated in one of the following three settings: Radboud University Medical Centre (Nijmegen), RMC Groot Klimmendaal (Arnhem), and community health center Buitenlust (Venray). Three intervention groups are planned in Nijmegen, three groups in Arnhem, and one group in Venray. Intervention groups consist of four to seven patients.

### Participants

Referred or otherwise interested patients are contacted by telephone by the primary investigator (YV) to provide detailed information about the content of the study, the informed consent procedure, and to answer any residual questions related to study participation. The primary informal caregiver of the patient is also asked to participate in the study when willing and available. When patients and caregivers express their willingness to participate and global screening of the inclusion and exclusion criteria indicates potential eligibility, appointments for visits with a rehabilitation physician and occupational therapist at the outpatient rehabilitation clinic of the Radboud University Medical Centre are made. During these visits the inclusion and exclusion criteria are thoroughly checked and the informed consent is signed.

Table 1 provides an overview of the applied inclusion and exclusion criteria. All patients should be at least 18 years old and have an established neuromuscular disease, preferably FSHD, IBM, or MM. They should suffer from chronic fatigue with a clear influence on their social participation. They should also be motivated for participation in the Energetic program and show ‘readiness to change’. The latter aspects are tested by motivational interviewing [15], without the use of any formal cut-off criteria. In addition, each patient has to be able to formulate at least three personalized goals with regard to social participation. Possible depressive symptoms and other psychiatric or cognitive symptoms are judged by the psychologist for their severity to determine whether patients can participate in the Energetic program.

**Table 1:** **Inclusion and exclusion criteria of the Energetic study**

**Inclusion criteria**	**Exclusion criteria**
1) Age 18 years and older	1) Cardiorespiratory problems that preclude participation in aerobic exercise training
2) Having one of the following chronic degenerative muscle diseases: facioscapulohumeral muscular dystrophy (FSHD), inclusion body myositis (IBM), mitochondrial myopathy, or other muscle disease	2) Severe cognitive impairment
3) Suffering from fatigue with an impact on daily occupation*	3) Depression or other psychiatric disorder, including addiction problems
4) Being motivated and ‘ready to change’*	4) Pregnancy
5) Being able to formulate at least three personalized participation goals*	5) Limited life expectancy (<5 years) due to known co-morbid condition
	6) Having participated in the Energetic (or similar) program before

*Assessed by Motivational Interviewing.

Upon inclusion, the following socio-demographic characteristics are registered in a questionnaire: age (years), gender (male/female), living together (yes/no), level of education (low, middle, high), employment status (yes/no), relationship with caregiver (partner/sibling/ child/friend). In addition, patients’ diagnoses and years of diagnoses are recorded. Patients’ self-reported knowledge, skills and confidence with regard to self-management of their disease are registered with the Patient Activation Measure (PAM) [16].

### Intervention

The Energetic program is administered in small groups of minimally four and maximally seven patients. Although it is a group program, aerobic exercise training and self-management strategies are individualized as much as possible. At all settings, the program is delivered by a physical therapist and an occupational therapist, both experienced with behavioural modification techniques, and consists of the following modules:

#### Aerobic exercise training

During four months (16 weeks) patients receive individually tailored aerobic exercise training from the physical therapist, amounting to sessions of 90 minutes with regular breaks as needed; during the first 9 weeks twice a week and during the last 7 weeks once weekly. Patients are expected to perform physical exercises themselves once and twice a week during these periods, respectively, so that that the overall physical training load amounts to three sessions of at least 30 minutes per week during the entire intervention period. Training intensity is aimed at 50-70% of the maximum heart rate, guided by a cardiac rhythm monitor mounted on the chest that is read out by a wrist watch. Fine tuning takes place based on the recovery rate. The training includes different exercises, such as walking on a treadmill, cycling on a home trainer, rowing, and using a cross trainer, depending on the preference and motor abilities of the individual.

#### Education about aerobic exercise

In three 60-minute sessions (weeks 2-4), patients are taught about general training principles by the physical therapist. During these sessions the following training principles are addressed: (1) attaining an adequate training stimulus, (2) the need to rest and recuperate, (3) designing and adhering to a feasible training program, and (4) prevention of overtraining and relapse. Besides emphasizing relapse prevention, patients are taught that relapse cannot entirely be prevented (e.g., after a flu) especially not when living with a chronic illness.

#### Self-management training in applying energy conservation strategies

During eight 90-minute sessions (weeks 2-8 and 10), training in energy conservation strategies is given by the occupational therapist. These sessions are based on an evidence-based program for patients with multiple sclerosis, including education, discussion, goal setting, practicing activities, and performing homework activities with the aim to teach patients how to integrate energy conservation strategies into their daily lives. [11]. The following energy conservation strategies are addressed:

(1) benefits of rests, (2) effectively communicating with the social environment, (3) applying principles of proper body mechanics and ergonomics, (4) adequately modifying the personal environment, (5) analyzing and adjusting individual activities, (6) setting priorities, (7) finding an activity-rest balance over the entire day and week, (8) setting short-term and long-term goals, and (9) careful planning of activities to achieve one’s personal goals.

#### Implementation and relapse prevention in daily life

During ten 60-minute sessions (weeks 5-13 and 16), the physical and occupational therapists support and empower the patients in implementation of the aerobic exercise training and energy conservation strategies into their daily lives until they are sufficiently able to maintain these skills. One session is specifically devoted to the preparation of healthy food and is provided by a dietician, while another session, provided by an occupational therapist, is specifically focused on self-management at work. Caregivers are involved in two sessions in which patients are encouraged to think about how to maintain their level of physical endurance after cessation of the intervention. During this module different types of exercise are explored and guided by sports trainers and physical therapists, such as swimming, Nordic walking, yoga, and body work-out. Patients are also stimulated to explore possibilities for exercising in their own environment and share these experiences with group members. The ultimate goal for each patient is to have a feasible home-exercise program encompassing at least three days a week. In order to check whether patients are indeed able to maintain their achieved level of physical activity and exercise, a booster session of two hours with the physical and occupational therapists is organized two months after the end of the intervention program to reinforce previously learned skills.

A typical day program lasts at least three hours and encompasses aerobic exercise training and one of the other modules, including a 45-minute break (see Table 2). Individual therapy compliance is recorded by the therapists with regard to both the attendance of training sessions, education sessions, and participation in homework assignments. Whenever applicable, patients are asked for their reasons for noncompliance or drop-out.

**Table 2:** **Typical day within the Energetic program**

**Time**	**Modules**
11.00 – 12.30	Aerobic exercise training
12.30 – 13.15	Break; including lunch and taking a shower
13.15 – 14.15/14.45	Group session
	a. Education on aerobic exercise training, or
	b. Fatigue management with energy conservation strategies, or
	c. Implementation and relapse prevention

Before the start of the study, the participating physical and occupational therapists in each clinical setting follow a specific training how to deliver the Energetic program and adopt a self-management approach. This training consists of theoretical education as well as teaching practical skills at Energetic group sessions. During the study period, two sessions are organized for all therapists to reinforce their learned skills, to reflect on the program, and to share information among one another. At any point during the study, therapists are stimulated to contact each other to share experiences and consult the primary investigator whenever necessary (YV).

Usual care consists of continuation of every-day life, which often means regular physical therapy or sometimes no intervention at all [17]. In both the experimental and control groups, all (additional) interventions are monitored during the study period. The consultation of healthcare and social support professionals is recorded with a healthcare-utilization questionnaire at every assessment focusing on the preceding months. Patients are not restricted in any activities.

### Outcome measures

All primary and secondary outcome measures for determining the effectiveness of the Energetic program are listed in Table 3. The primary outcome is the patient’s self-rated performance in three to five self-identified problematic daily occupations assessed with the Canadian Occupational Performance Measure (COPM-performance) [18]. The COPM is a semi-structured interview designed to help patients to identify problems in their occupational performance using a structured scoring method. The patient has to put forward a minimum of three and maximum of five problematic daily occupations. These occupations are subsequently rated on a 10-point scale for perceived performance capacity (1 = not able at all, 10 = perfectly able). Previous studies have shown that the COPM is a valid and responsive instrument to assess self-care, productivity and leisure and patients’ perspectives related to these activities [19,20].

**Table 3:** **Outcome measures**

**Participants**	**Outcome**	**Outcome measure**
**Patients**	**Primary outcome measure**	
	Social participation – performance of occupations	Canadian Occupational Performance Measure, performance score (COPM-performance) [18-20]
	**Secondary outcome measures**	
	Social participation- satisfaction with occupational performance	Canadian Occupational Performance Measure, satisfaction score (COPM-satisfaction) [18-20]
	Fatigue impact and severity	Checklist Individual Strength - subscale Fatigue (CIS-Fatigue) [21]
	Physical endurance	6-Minute Walking Test [22]
	Activity engagement	Activity Card Sort (ACS) [23,24]
	Self-efficacy	General Self-Efficacy Scale [26]
	Mood	Hospital Anxiety and Depression Scale (HADS) [25]
	Resource utilization	Self-developed resource utilization questionnaire
	Health-related quality of life	Health-related Quality of Life: Short Form 36 (SF-36) [28]
**Caregiver(s)**	Perceived caregiver burden	Zarit Burden Interview (ZBI) [27]
	Objective caregiver burden	Self-developed scale for objective care burden questionnaire; amount of care (hours)

Secondary measures of effectiveness include the COPM-satisfaction score (1 = not satisfied at all, 10 = extremely satisfied) [18-20], Checklist Individual Strength - subscale fatigue (CIS-Fatigue) [21], physical endurance (6-Minute Walking Test) [22], activity engagement (Activity Card Sort) [23,24], mood (Hospital Anxiety and Depression Scale) [25], and self-efficacy (General Self-Efficacy Scale) [26]. At the level of the caregiver, secondary outcomes are perceived caregiver burden (Zarit Burden Interview) [27] and objective caregiver burden (Objective Care Burden Questionnaire; a self developed scale for amount of care (hours)).

Outcome measures in the economic evaluation are costs (direct health costs and indirect societal costs) and quality adjusted life years (QALY). The quality of the health status of the patients is based on the Short Form-36 Health Survey (SF-36) [28] and costs are based on a self-developed resource utilization questionnaire.

### Sample size calculation

The primary outcome is the change in COPM-performance score at the end of the program (T1) since baseline (T0). A conservative estimate of the improvement of the Energetic group compared to usual care is set at 2.0 and the standard deviation of the change in each group at 1.7. These numbers are based on the results of our pilot study with 13 participants [14]. Based on these assumptions, 13 participants are needed in each group to obtain a power of 80% (two sided t-test set 5%). Taking into account a drop-out rate of 10%, a total of 30 participants would be required. However, to meet the required estimates for determining the cost-effectiveness, our aim is to include 50 patients.

### Statistical analysis

The primary variable for effectiveness will be analyzed in a covariance model with the COPM-performance scores at the end of the program (T1) as dependent variable. The baseline COPM-performance (T0) and the minimization factors will be covariates. Two-sided 95% confidence intervals will be presented. Data will be analyzed following the principle of intention-to-treat. Similarly, the secondary variables will be evaluated.

The economic evaluation is based on the principles of a cost-effectiveness (utility) analysis from a societal perspective. The primary incremental cost-effectiveness ratio is the cost per QALY gained (ICER) based on SF-6D utilities [29]. Uncertainty surrounding this ICER will be determined using the bootstrap method. A cost-effectiveness acceptability curve will be derived that is able to evaluate the probability that ‘Energetic’ is efficient against different thresholds (Willingness To Pay) for a QALY.

## Discussion

To the best of our knowledge, the Energetic study is the first randomized controlled trial to evaluate the effectiveness and cost-effectiveness of a combined physical and self-management group training program for improving social participation, physical endurance and alleviating fatigue in patients with neuromuscular diseases (NMDs). We expect that this study will generate new insights in (cost-) effective rehabilitation strategies for these incurable conditions.

The current study includes patients with various forms of NMD, but specifically focuses on patients with FSHD, IBM and MM. These are all chronic degenerative muscle diseases with a slowly progressive character. The commonality among these disorders is the struggle that patients experience to manage their lives with chronic fatigue and reduced physical capacity. Although the study is powered to establish (cost-)effectiveness across the different diagnoses, an attempt will be made to perform subgroup analyses, using diagnosis as a between-subjects factor, to detect trends in differences in effect sizes and therapy compliance between groups. In addition, diversity will be addressed by examining the influence of socio-demographic characteristics on effect size and therapy compliance.

As a primary outcome, we chose the COPM because this measure potentially fits best with the self-management and participation goals of the Energetic program. The COPM assesses the perceived performance of daily activities as well as the individual satisfaction with these activities, and has shown to be a sensitive and clinically relevant outcome in many other rehabilitation studies [30-33]. In addition, a wide range of secondary outcome measures is used to fully examine and understand the impact of the Energetic program on the lives of patients with NMD and their caregivers. The economic evaluation serves to underscore the financial and societal impact of the program and to secure future reimbursement by health insurances.

We purposively included three clinical settings for a first implementation of the Energetic program: besides a university medical center, we selected a regional rehabilitation center and a community health center. This choice allows us to evaluate barriers and facilitators of implementation in each of these settings, which will help future dissemination of the Energetic program across other health institutions. To this end, we will monitor protocol adherence by therapists using a logbook, organize booster sessions for therapists during the study, and organize a group meeting with all therapists for process evaluation after the study period.

## Abbreviations

FSHD: Facioscapulohumeral muscular dystrophy
IBM: Inclusion body myositis
MM: Mitochondrial myopathies
NMD: Neuromuscular diseases
CIS: Checklist Individual Strength
COPM: Canadian Occupational Performance Measure
HRQoL: Health-related quality of life
QALY: Quality adjusted life years
